# Patriotism in British Hospitals

**Published:** 1917-02-10

**Authors:** 


### PATRIOTISM IN BRITISH HOSPITALS.

### Two London Medical Schools.

### Westminster Hospital.

THE outbreak of war affected the medical staff of this hospital to an appreciable extent. Four members of the senior staff took up appointments abroad; one of the physicians was appointed consulting physician to H.M. Forces in the Eastern Mediterranean, and another is in charge of a base hospital in France. The senior surgeon, who has since died from illness contracted in Egypt, was appointed consulting surgeon to H.M. Forces in Egypt, and another consulting surgeon was dispatched to the Eastern Mediterranean. Of the remaining members of the staff, seven were attached to Base Hospital No. 4, and one of them to King George Hospital as well.

In addition to this every member of the staff is attached to several of the smaller hospitals, principally for officers, which have been established in many private houses. The medical registrar accepted service in the Royal Navy at the commencement of the war, and his place has ever since been vacant, as is also that of surgical registrar. As regards the resident staff it has been reduced to two qualified men in place of eight. Fortunately, several capable senior students were able to render material aid though not yet fully qualified. More than thirty of the past and present qualified students accepted temporary commissions in the R.A.M.C., and two partly qualified are also serving.

### King's College Hospital.

SINCE the commencement of the war the major portion of King's has been occupied by the 4th London General Hospital (T.), and the military wards have been filled with wounded soldiers. Many members of the staff hold commissions in the Medical Services, five being in the Navy and twenty-four in the Army. It will thus be seen that the carrying on of medical education presented great difficulties; but by careful organisation these difficulties have been overcome, and the teaching of the school has been continued with the same thoroughness which has always been one of its chief characteristics.

The information respecting the war service of past and present members of the medical school is incomplete, but so far as present information goes, thirty-three are serving in the Medical Services of the Navy and one hundred and seventy-eight in the Army. Junior students holding commissions or serving in the ranks of the combatant Forces number thirty-one.

The students who are continuing their final studies at the hospital (with the exception of those from the Dominions) attested under Lord Derby's Scheme, and are enrolled in the Medical Unit of the O.T.C.

HONOURS.

*K.C.B.; Legion of Honour, Grand Officer; Commandeur de l'Ordre de Leopold.*—Sir Arthur Thomas Sloggett, C.M.G., K.H.S., Director-General Army Medical Service.

*K.C.M.G.*—Sir William Watson Cheyne, Bart., C.B., Consulting Surgeon and Surgeon-General, Royal Navy; Colonel M. P. C. Holt, C.B., D.S.O., A.M.S.

*C.B.*—Colonel F. F. Burghard, A.M.S.; Surgeon-General W. H. Norman, R.N.

*C.M.G.*—Lieutenant-Colonel H. A. Bray, R.A.M.C.; Lieutenant-Colonel A. H. Lister, R.A.M.C.; Colonel B. J. Newmarch, Australian A.M.C.; Lieutenant-Colonel F. S. Penny, R.A.M.C.

*Military Cross.*—Captain E. W. Carrington, R.A.M.C.; Captain C. F. Hacker, R.A.M.C.; Captain S. H. Smith, R.A.M.C.; Captain F. T. Turner, R.A.M.C.; Captain H. S. Turner, R.A.M.C.; Lieutenant J. R. Waddy, R.A.M.C.

*D.S.O.*—Surgeon-Major R. M. Cowie, 1st Life Guards; Major G. W. G. Hughes, R.A.M.C.; Major (temporary Lieutenant-Colonel) A. C. Osburn, R.A.M.C.; Lieutenant-Colonel H. S. Roch, R.A.M.C.; Captain W. C. Smales, R.A.M.C.; Lieutenant-Colonel W. M. B. Sparkes, R.A.M.C.; Major F. A. Stephens, R.A.M.C.

*Mentioned in Dispatches.*—Major C. H. Barber, I.M.S.; Lieutenant-Colonel H. A. Bray, C.M.G., R.A.M.C.; Colonel F. F. Burghard, C.B., A.M.S.; Captain E. W. Carrington, M.C., R.A.M.C.; Surgeon-Major R. M. Cowie, D.S.O., 1st Life Guards; E. S. Crispin, Medical Department, Sudan Government; Captain H. B. Day, R.A.M.C.; Major (temporary Lieutenant-Colonel) N. E. Dunkerton, R.A.M.C.; Captain R. H. C. Gompertz, R.A.M.C.; Major H. E. Griffiths, R.A.M.C.; Captain C. F. Hacker, R.A.M.C.; Major G. W. G. Hughes, D.S.O., R.A.M.C.; Colonel B. J. Newmarch, C.M.G., Australian A.M.C.; Major (temporary Lieutenant-Colonel) A. C. Osburn, D.S.O., R.A.M.C.; Captain K. Playfair, R.A.M.C.; Lieutenant-Colonel H. S. Roch, R.A.M.C.; Surgeon-General Sir A. T. Sloggett, K.C.B., K.H.S., C.M.G.; Captain W. C. Smales, D.S.O., R.A.M.C.; Lieutenant-Colonel W. M. B. Sparkes, D.S.O., R.A.M.C.; Lieutenant J. R. Waddy, M.C., R.A.M.C.; Major J. C. Webb, R.A.M.C.; Captain T. H. Whittington.

KILLED IN ACTION.

Sydney Howard Hodges, B.Sc., Lieutenant, Royal Fusiliers; John Raymond Waddy, B.A., Lieutenant, R.A.M.C.; Edward Worrell Carrington, M.A., M.B., B.Ch., Captain, R.A.M.C.; Albert William Buchan Carless, Second Lieutenant, Middlesex Regiment; Sydney Clark, Captain, R.A.M.C.

